# A comparative analysis of carcass traits and meat quality between Tibetan sheep and their three-way crossbred sheep

**DOI:** 10.3389/fnut.2025.1620180

**Published:** 2025-07-16

**Authors:** Yu Qiao, Deming Yang, Jishun Tang, Wei Zhang, Sheng Chen, Wenqiao Hui

**Affiliations:** ^1^Anhui Province Key Laboratory of Livestock and Poultry Product Safety Engineering, Institute of Animal Science and Veterinary Medicine, Anhui Academy of Agricultural Sciences, Hefei, China; ^2^Qinghai Yangsen Agricultural and Livestock Ecological Co., Ltd., Gonghe, China

**Keywords:** crossbred lamb, Tibetan sheep, muscle development, meat flavor, differentially expressed genes

## Abstract

**Introduction:**

To improve meat yield and nutritional quality of Tibetan sheep of the Qinghai-Tibet Plateau, Australian White and Small-tailed Han rams were introduced to crossbred with Tibetan sheep (Oura-type, O) ewes which possessed genetic homogeneity. The aim of this study was to compare growth conformation, meat quality, and further identified related candidate genes between Tibetan sheep and their three-way crossbreds (25% Australian White × 25% Small-tailed Han × 50% Oura-type Tibetan, AHO).

**Methods:**

Fifteen 5.5-month-old male lambs (8 O and 7 AHO crossbred) were raised for 5.5 months. We compared body conformation, carcass traits, meat quality (pH, color, cooking loss), nutrient composition (amino acids, fatty acids, ribonucleotides) in *longissimus thoracis et lumborum* (LTL) muscle, and analyzed differentially expressed genes through transcriptomics.

**Results:**

Our findings indicated that 5.5-month-old AHO lambs exhibited significantly superior body conformation and carcass traits (*p* < 0.05), including live weight, cannon circumference, carcass weight, and loin eye area. Their LTL muscle contained higher levels of PUFA, n-6 PUFA, and a greater PUFA/SFA ratio along with the content of glutamic acid and methionine (*p* < 0.05), and an increased trend for inosine monophosphate (*p* = 0.072) in the AHO lambs than O groups. Transcriptomic analysis identified candidate genes including *CSRP3*, *ANKRD1*, *IFRD1*, *PPARGC1A*, and *AMPD3*, which are differentially expressed and associated with muscle development and meat flavor.

**Conclusion:**

The study revealed that AHO lambs demonstrated better growth and meat quality, with identified candidate genes for these traits. These findings provide practical strategies for enhancing Tibetan lamb meat quality through crossbreeding optimization in high-altitude regions.

## 1 Introduction

Tibetan sheep is a native sheep breed mainly produced in Qinghai-Tibet plateau. It is also one of the important livestock resources in China (1). Due to long-term natural selection, Tibetan sheep at high altitudes have attained a distinctive suite of physiological adaptations to high-altitude hypoxia. However, their growth performance is limited by the extreme conditions of the Tibetan plateau, resulting in small size, slow growth rate, and low slaughter rate, thereby limiting lamb production (2). As living standards continue to rise, consumer demand for premium-quality lamb in sufficient quantities has grown significantly (3). Therefore, it is crucial to breed Tibetan sheep that combine excellent growth performance with high meat quality in order to meet the needs of the local animal husbandry and meat product industries.

Crossbreeding enhances sheep growth performance and meat quality (4). However, there has been rare exploration of crossbreeding programs involving Tibetan sheep and exotic breeds until now. The Australian White sheep breed is known for its robust stress resistance, quick growth rate, and ease of management. Crossbreeding with local sheep breeds is deemed effective for enhancing meat quality and production efficiency (5). Small-tailed Han sheep is an excellent local breed in China, with good-flavored meat and high prolificacy (6). Based on this, a three-way cross (25% Australian White × 25% Small-tailed Han × 50% Oura-type Tibetan) program is performed to explore whether hybridization with the foreign commercial breeds can improve the growth conformation and meat quality of Tibetan sheep.

Meat quality encompasses appearance, physical attributes, and flavor. Meat flavor, determined by fatty acid profiles, amino acids and ribonucleotides, is a critical driver of consumer preference. Various non-volatile substances influence meat quality and flavor, with fatty acids, amino acids, and ribonucleotides in mutton specifically contributing to its distinct flavors (7). Mutton’s unique flavor is primarily influenced by its lipid content, with the ratio of fatty acids, especially unsaturated ones, being crucial in defining its taste profile (8). Excessive saturated fatty acid consumption is associated with higher risks of coronary heart disease, diabetes, and obesity (4). Monounsaturated (MUFA) and polyunsaturated fatty acids (PUFA) can lower triglyceride and cholesterol levels, thereby reducing cardiovascular disease risk and enhancing immunity (9). Amino acids generally have complex taste profiles, encompassing sour, salty, umami, sweet, and bitter flavors, with natural amino acids predominantly exhibiting bitterness due to their structural characteristics. Flavor amino acids, including glutamic acid, glycine, alanine, and aspartic acid, are key determinants of food taste (10). Ribonucleotides like inosine-5′-monophosphate (IMP) and guanosine-5′-monophosphate (GMP) are key umami compounds in meat, contributing to its flavor enhancement. Hypoxanthine and inosine, which are degradation products of IMP during aging, may influence meat flavor due to their bitter taste (11).

Omics analysis techniques are increasingly being used in complex trait analysis to explore the potential molecular mechanisms underlying sheep hybridization (12). Transcriptomics can identify many DEGs, which are then screened and analyzed to identify key gene targets (13). Based on this, we performed transcriptomic analysis on the *longissimus thoracis et lumborum* (LTL) muscle of the F1 AHO and O lamb in order to reveal the differentially expressed genes in the muscle tissues between the AHO group and the O group.

To date, there has been limited research comparing the growth performance and meat quality characteristics of AHO crossbred sheep with those of Tibetan sheep. This study compared the differences in body conformation, carcass traits, meat quality traits, fatty acid and amino acid composition, and ribonucleotide content between two groups of sheep. Furthermore, transcriptomic analysis was performed to identify candidate genes associated with muscle development and meat quality. The results of this study may promote an understanding of the differences between crossbred sheep and Tibetan sheep and offer a theoretical foundation for improving meat quality in sheep.

## 2 Materials and methods

### 2.1 Animals and experimental design

The animal study protocol was approved by the Animal Welfare Committee of the Anhui Academy of Agricultural Sciences (AAAS2023-01). Eight male newborn Oura-type Tibetan F1 lambs (O) and seven male newborn three-way crossbreed AHO F1 lambs (25% Australian White × 25% Small-tail Han × 50% Oura-type Tibetan) were selected under the same natural grassland (Gonghe County, Qinghai Province, China) grazing conditions. The initial average body weight of O and AHO lambs was 3.85 ± 0.42 kg and 4.32 ± 0.11 kg, respectively, with no significant difference between groups (*p* > 0.05). All animals were clinically healthy, having received routine vaccinations and antiparasitic treatments prior to the trial. After a 2-week adjustment period (lambs completely transitioned from breast milk to pasture), the lambs were grazed on natural pasture from 07:00 to 18:00 daily, with supplemental concentrate feeding (300 g/lamb/day, concentrate feeding nutritional composition, moisture ≤14%, crude protein ≥15%, crude fiber ≤25%, crude ash ≤12%, calcium 0.5–1.5%, phosphorus ≥0.3%, salt 0.3–2.0%, lysine ≥0.4%), and the fattening phase lasted 5.5 months. The body weight of lambs in both the O and AHO groups was recorded at 5.5 months old.

### 2.2 Body conformation and carcass traits

Upon completion of the 5.5 months fattening period, lambs were slaughtered. Body conformation traits such as body height, body length, chest circumference, and cannon circumference were measured for the O and AHO lambs, as outlined in Yang et al. (14). The traits were determined and recorded by the same person to minimize random errors in this experiment. After an overnight fast with unrestricted water access, the live weight (fasted) of all lambs were measured. All lambs were euthanized by electric shock prior to exsanguination and slaughter. After evisceration, the weight of the carcass and tare were measured, respectively. The dressing percentage was determined by dividing the carcass weight by the fasted live weight. Following the full dissection of the carcass, the organ indices (organ weight relative to fasted live weight) of the heart, liver, spleen, lungs, kidneys, pancreas, and thymus were documented. The loin eye contour between the 12th and 13th ribs on the left side of each carcass was traced onto paper, and the area was measured using a scaled grid. After slaughter, muscle samples are collected and subsequently stored at −80°C until required for analyses. Before determining composition of fatty acids and amino acids of LTL muscle, the samples were removed from the −80°C refrigerator, placed in sealed bags, and slowly thawed at 4°C for 24 h to ensure that the final stable content of metabolites in the samples is detected.

### 2.3 Meat quality determination

After slaughter, carcasses were immediately transferred to a controlled aging chamber (0–2°C, 85% relative humidity, 0.5 m/s air velocity) for 24 h. During this period, the pH of the LTL muscle was measured at 45 min and 24 h postmortem using a portable pH meter (Testo 205, Testo Instrument Co. Ltd., Germany) supplied with electrodes with internal temperature sensor, with pH resolution of 0.01, and automatic calibration with two-point buffer set standard for pH 4.01 and 7.01. The color of the fresh LTL muscle was measured following a 45-min rest at 4°C. A color difference reader (CR-10, Minolta, Japan) was used to measure three positions on the cut surface of each sample. The illuminant, standard observer and aperture used were: light source (illuminant) - D65 (daylight at noon); degree of observer - 2° and size of measuring port - 8 mm. The average of the three measurements was recorded as a pH (or color) coordinate value for each sample. The percentage cooking loss of the meat pieces, which were after aging, was determined by measuring the weight difference before and after cooking, as described by Jiao et al. (15). The protein and fat content in the samples was determined using AOAC12 methods (2005) (16). The protein content (%) in the LTL muscle was measured using a carbon/nitrogen analyzer, while total lipid content (%) was determined via the ether extraction method (AOAC 920.39).

### 2.4 Amino acids composition

Amino acids were analyzed using a SYKAM S-433D amino acid analyzer (Germany). A 0.15 g sample of LTL muscle was placed in a hydrolysis tube with 15 mL of 6 M hydrochloric acid and 4 drops of phenol solution, then subjected to a 5-min freeze and vacuum treatment. Nitrogen was introduced, and the mixture underwent hydrolysis for 22 h before cooling to room temperature. The hydrolysate was diluted with 1 mL of ammonium citrate buffer (pH 2.2), collected as filtrate, vacuum-dried, and filtered through a 0.22-μm membrane. The hydrolysate was filtered to maintain a consistent volume in a 50 mL container. Amino acids were eluted with a sodium buffer using an ion-exchange chromatographic column (650–0042, Bodenheim, Germany). The compounds formed upon reaction with ninhydrin were concurrently measured at 570 nm and 440 nm wavelengths. The detailed procedure was conducted according to the Chinese standards for the measurement of amino acid composition within foods (GB 5009.168–2016).

### 2.5 Fatty acids composition

A 3 g meat sample was powdered, homogenized with a chloroform-methanol mixture (2:1, v/v), and the solvent was evaporated. The internal standard, methyl non-adecanoate was added during extraction process. A reflux condenser was attached to the fat extract after adding 8 mL of 2% sodium hydroxide methanol solution for saponification and methyl esterification of fatty acids. N-heptane (3 mL) was added and shaken for 2 min. Subsequently, 2 mL of saturated sodium chloride solution was added, followed by the absorption of the upper layer. Anhydrous sodium sulfate was then introduced and thoroughly mixed. The upper solution was extracted into an injection vial for gas chromatographic analysis using an Agilent 7890A system (California, CA, USA). The experimental setup included a chromatographic column measuring 100 m × 0.25 mm × 0.2 μm. The inlet and detector temperatures were set at 250°C and 280°C, respectively. High-purity helium was used as the carrier gas at a flow rate of 1 mL/min. The injection volume was 1.0 μL with a split ratio of 20:1. The heating program was following the previous study (17). Fatty acids were identified by comparing retention times and quantified based on peak areas. The absolute quantity of each fatty acid was represented as a percentage of the total fatty acid methyl esters. MUFA represent the total of all monounsaturated fatty acids, while PUFA denote the total of all polyunsaturated fatty acids. n-3 PUFA consists of C18 3n-3, C20 3n-3, C20 5n-3, and C22 6n-3, while n-6 PUFA includes C18 2n-6c, C18 3n-6, and C20 3n-6. Nutritional indices, including the index of atherogenicity (IA), index of thrombogenicity (IT), and hypocholesterolemic/hypercholesterolemic ratio (H/H), were used to assess the nutritional quality of fatty acids (18, 19). The indices were computed as follows:


$$\mathrm{IA}=\frac{C12:0+(4\times C14:0)+C16:0}{\text{PUFA}+\text{MUFA}}$$

$$\mathrm{IT}=\frac{C14:0+C16:0+C18:0}{\left(0.5\times \text{MUFA})+(0.5\times n-6)+(3\times n-3)+(\frac{n-3}{n-6}\right)}$$

$$H/H=\frac{C18:1+\text{ΣPUFA}}{C12:0+C14:0+C16:0}$$

### 2.6 Ribonucleotide analysis

The LC–MS/MS method was employed to quantify IMP, GMP, 6-hypoxanthine, and inosine in muscle samples. Approximately 200 mg of fully ground sample powder was placed in a 2 mL centrifuge tube. An extraction solution (1.5 mL) composed of methanol and water in a 10:90 volume ratio with 0.1% formic acid was subsequently added. The mixture was rotated and mixed, and the sample was ultrasonically extracted for 30 min. Then, 400 μL of n-hexane was added. Centrifuge the tube at 12,000 rpm for 5 min, dilute the middle liquid phase 100-fold, filter through a 0.22 μm organic membrane, and transfer to a sample vial for LC–MS analysis. The liquid phase parameters are as follows: a Thermo HYPERSIL GOLD C18 chromatographic column (2.1 × 100 mm, 3 μm) is used with a column temperature of 35°C. The injection volume is 5 μL and the flow rate is set at 0.3 mL/min. The mobile phase consists of 0.1% formic acid in water (phase A) and acetonitrile (phase B). The elution gradient is as follows: from 0 to 2 min, phase A is 90%; from 2 to 6 min, phase A decreases to 10%; from 6 to 8 min, phase A remains at 10%; from 8 to 8.1 min, phase A increases back to 90%; and from 8.1 to 10 min, phase A is maintained at 90%. The mass spectrometry parameters include an ESI (Turbo Spray) ion source with negative polarity, a spray voltage of −4,500 V, a curtain gas pressure of 30 psi, a collision gas pressure of 9 psi, and a nebulization temperature of 550°C. The Multiple Reaction Monitoring method was used for quantitative acquisition of data from mass spectrometry.

### 2.7 RNA-seq data analysis

Total RNA was extracted from the LTL muscle tissues using the RNAiso Plus according to the manufacturer’s instructions (Thermo Fisher Scientific). The RNA samples were assessed for integrity, concentration, and purity. Following the RNA-Seq sample preparation kit instructions (Illumina, San Diego, USA), the purified mRNA was fragmented and reverse transcribed to generate the final cDNA library. The libraries were sequenced using the Illumina Novaseq™ 6,000 platform by LC Bio Technology Co., Ltd. in Hangzhou, China. Bioinformatic analysis utilized data produced by the Illumina platform. Gene differential expression analysis was completed by DESeq2 software and edgeR. The genes with the parameter of false discovery rate (FDR) below 0.05 and absolute fold change (FC) ≥ 2 were considered DEGs. Then, all DEGs were mapped to the Gene Ontology (GO) database^1^ and Kyoto Encyclopedia of Genes and Genomes database (KEGG)^2^ for recognizing the main biological functions.

### 2.8 Reverse transcription-quantitative PCR

RNA-reverse transcription and quantitative PCR were performed as described in our previous publications (13). The primer sequences for the genes analyzed in this study are provided in Supplementary Table S1. The Ct values of target DEGs were normalized based on the Ct values of GAPDH, which were found to be stably expressed in different conditions. Subsequently, the 2^-ΔΔCt^ method was used to determine the relative mRNA expression of the target DEGs. All trial samples had three biological replicates.

### 2.9 Statistical analysis

Data were statistically analyzed using SPSS software (Version 23.0, SPSS Inc., Chicago, IL, USA), with results expressed as means ± standard error of the mean (SEM). A Student’s t-test or Mann–Whitney U-test was used to compare the two groups. A *p*-value below 0.05 signifies a statistically significant difference. All figures were made using the OmicStudio tools,^3^ GraphPad Prism (Version 9.0, Graph Pad Software Inc., San Diego, CA, USA) and Adobe Photoshop (Adobe Photoshop CC 2018, Inc., San Jose, California, USA).

## 3 Results

### 3.1 The body conformation traits and carcass traits

First, we evaluated and compared the body conformation traits of lambs within two groups at 5.5 months of age, as shown in Table 1. The AHO group exhibited significantly greater live weight, body length, and cannon circumference compared to the O group (*p* < 0.05). However, the body height and chest circumference were not significantly different across the AHO and O groups (*p* > 0.05). Carcass traits were assessed post-sacrifice, as detailed in Table 2. The AHO group exhibited a significantly higher carcass weight compare to the O group (*p* < 0.05), with a tendency for increased tare weight (*p* = 0.091). There was no significant difference in dressing percentage between the O and AHO groups (*p* > 0.05). The AHO lamb exhibited a significantly larger LTL muscle loin eye area compared to the O lamb (*p* < 0.05). For organ weight and indices of lamb, the weight and index of spleen, and the lung weight were higher in the AHO group than those in the O group (*p* < 0.05). There were no significant differences in the weights and indices of the heart, liver, kidney, pancreas, and thymus between the two groups (*p* > 0.05). These results showed that AHO lamb showed better body conformation and carcass traits than that of O lamb.

**Table 1:** Body conformation traits of 5.5-month-old O and AHO lambs.

Indexes	O	AHO	*p*-value
Live weight (kg)	34.53 ± 0.18^b^	38.29 ± 1.15^a^	0.030
Body height (cm)	67.80 ± 0.98	68.08 ± 0.56	0.801
Body length (cm)	66.57 ± 0.82	70.98 ± 1.94	0.099
Chest circumference (cm)	82.30 ± 0.66	86.74 ± 2.36	0.149
Cannon circumference (cm)	7.43 ± 0.19^b^	8.00 ± 0.11^a^	0.029

AHO: Australian White × Small-tailed Han × Oura-type Tibetan three-way crossbred sheep; O: Oura-type Tibetan sheep; ^a,b^ different letters indicate significant differences between two groups (*p* < 0.05). Data were represented as means ± SEM.

**Table 2:** The carcass traits of 5.5-month-old O and AHO lambs.

Item	O	AHO	*p*-value
Carcass weight (kg)	17.87 ± 0.26^b^	19.65 ± 0.67^a^	0.047
Dressing percentage (%)	50.02 ± 0.50	50.46 ± 0.16	0.436
Tare weight (kg)	2.50 ± 0.07	2.81 ± 0.13	0.091
Loin eye area (cm^2^)	19.11 ± 0.74^b^	25.80 ± 1.25^a^	0.004
Heart index (%)	0.44 ± 0.02	0.45 ± 0.03	0.874
Liver index (%)	1.55 ± 0.12	1.60 ± 0.10	0.732
Spleen index (%)	0.11 ± 0.00^b^	0.13 ± 0.00^a^	0.001
Lung index (%)	0.96 ± 0.02	1.06 ± 0.07	0.257
Kidney index (%)	0.27 ± 0.03	0.25 ± 0.01	0.572
Pancreas index (%)	0.07 ± 0.01	0.08 ± 0.01	0.372
Thymus index (%)	0.16 ± 0.02	0.12 ± 0.01	0.128

AHO: Australian White × Small-tailed Han × Oura-type Tibetan three-way crossbred sheep; O: Oura-type Tibetan sheep; ^a,b^ different letters indicate significant differences between two groups (P < 0.05). Data were represented as means ± SEM.

### 3.2 Meat quality characteristics of the LTL muscle

Given that the crossbred AHO show better growth performance than O lamb, we were then headed to see whether the crossbreeding influences the meat quality including meat appearance and physical properties. The characteristics of pH, objective color (a*, b*, and L* values), crude protein, total lipid content, and cooking loss of the LTL muscle were evaluated after the lambs were sacrificed. The meat pH (45 min and 24 h) and the instrumental color values of a*, b*, and L* were not significantly different across the AHO and O groups (*p* > 0.05). There were no significant differences in cooking loss, crude protein, and total lipid content between the AHO and O meat groups (*p* > 0.05) (Table 3).

**Table 3:** The meat quality characteristics in LTL muscle of O and AHO lambs.

Item	O	AHO	*p*-value
pH (45 min)	6.25 ± 0.10	6.38 ± 0.13	0.457
pH (24 h)	5.88 ± 0.16	5.87 ± 0.19	0.970
Meat color a*	8.91 ± 1.18	8.97 ± 0.75	0.962
Meat color b*	6.59 ± 0.86	7.48 ± 0.61	0.413
Meat color L*	28.23 ± 1.76	30.19 ± 1.33	0.394
Cooking loss (%)	43.85 ± 1.73	44.62 ± 0.71	0.666
Crude protein (%)	19.43 ± 0.62	19.36 ± 0.43	0.932
Total lipid content (%)	5.53 ± 0.07	3.98 ± 0.68	0.104

AHO: Australian White × Small-tailed Han × Oura-type Tibetan three-way crossbred sheep; O: Oura-type Tibetan sheep; ^a,b^ different letters indicate significant differences between two groups (*p* < 0.05). Data were represented as means ± SEM.

### 3.3 Fatty acid, amino acid composition and ribonucleotides content in the LTL muscle

Recognizing meat flavor as a crucial aspect of meat quality and a significant factor in consumer purchasing decisions, we analyzed and compared the fatty acids, amino acids, and ribonucleotides composition and content in lambs from the two groups. First, we detected the LTL muscle fatty acid composition of lamb from the two groups. Table 4 indicates a significant increase in capric acid (C10:0), dihomo-*γ*-linolenic acid (C20:3n-6), and nervonic acid (C24:1 n-9) levels in the AHO group compared to the O group (*p* < 0.05). The AHO group exhibited a tendency for reduced oleic acid (C18:1n-9c) levels compared to the O group (*p* = 0.065). In the AHO group, levels of linoleic acid (C18:2n-6c), *α*-linolenic acid (C18:3n-3), γ-linolenic acid (C18:3n-6), and eicosadienoic acid (C20:2) tended to be higher compared to the O group, with *p*-values of 0.089, 0.086, and 0.069, respectively. No significant differences were observed in the levels of other fatty acids between the O and AHO groups (*p* > 0.05). The AHO group exhibited a tendency for reduced MUFA content compared to the O group (*p* = 0.056). PUFA and n-6 PUFA levels tended to increase compared to the O group (*p* = 0.082 and 0.086, respectively). There was no significant difference in n-3 PUFA content between the O and AHO groups (*p* > 0.05). The PUFA/SFA ratio tended to be higher in the AHO group than in the O group (*p* = 0.098). The IT value tended to decrease in the AHO group compared to the O group (*p* = 0.074). The O and AHO groups showed no significant differences in the n-6/n-3 ratio, IA value, and H/H rate (*p* > 0.05).

**Table 4:** The fatty acid profile in the LTL muscle of O and AHO lambs.

Fatty acid content (mg/100 g)	O	AHO	*p*-value
C4:0	0.07 ± 0.01	0.11 ± 0.02	0.168
C8:0	0.20 ± 0.06	0.22 ± 0.05	0.852
C10:0	1.20 ± 0.09^b^	1.51 ± 0.08^a^	0.034
C12:0	1.29 ± 0.27	1.96 ± 0.46	0.280
C13:0	0.10 ± 0.01	0.11 ± 0.03	0.904
C14:0	28.05 ± 2.50	29.46 ± 3.95	0.786
C14:1n-5	0.85 ± 0.09	0.92 ± 0.14	0.726
C15:0	2.86 ± 0.35	2.83 ± 0.31	0.948
C16:0	286.00 ± 6.18	291.20 ± 4.83	0.522
C16:1n-7	16.4 ± 0.78	14.36 ± 0.95	0.153
C17:0	9.57 ± 1.72	9.51 ± 0.78	0.973
C18:0	163.25 ± 10.05	167.00 ± 7.16	0.764
C18:1n-9c	439.50 ± 3.80	416.80 ± 8.69	0.065
C18:2n-6c	35.30 ± 3.06	45.24 ± 3.75	0.089
C18:3n-3	1.03 ± 0.13	1.38 ± 0.21	0.055
C18:3n-6	0.42 ± 0.06	0.54 ± 0.03	0.088
C20:0	0.64 ± 0.07	0.70 ± 0.06	0.542
C20:1	0.59 ± 0.03	0.71 ± 0.10	0.313
C20:2	0.23 ± 0.03	0.34 ± 0.04	0.068
C20:3n-3	0.19 ± 0.01	0.20 ± 0.01	0.451
C20:3n-6	0.65 ± 0.05	0.88 ± 0.08	0.048
C20:5n-3	2.22 ± 0.03	2.29 ± 0.05	0.329
C22:0	0.11 ± 0.01	0.13 ± 0.01	0.058
C22:1n-9	0.22 ± 0.02	0.20 ± 0.02	0.387
C22:6n-3	0.20 ± 0.04	0.25 ± 0.05	0.485
C23:0	8.64 ± 0.86	10.87 ± 0.98	0.142
C24:0	0.14 ± 0.01	0.17 ± 0.01	0.242
C24:1n-9	0.14 ± 0.03^b^	0.26 ± 0.03^a^	0.033
SFA	502.12 ± 3.02	515.75 ± 5.14	0.071
MUFA	457.71 ± 3.24	433.24 ± 9.11	0.056
PUFA	40.23 ± 3.29	51.12 ± 3.98	0.082
n-6	36.37 ± 3.15	46.66 ± 3.83	0.086
n-3	3.63 ± 0.21	4.11 ± 0.31	0.261
n-6/n-3	10.03 ± 0.76	11.47 ± 0.94	0.290
PUFA/SFA	0.72 ± 0.06	0.90 ± 0.06	0.098
AI	8.02 ± 0.27	8.49 ± 0.41	0.394
TI	92.85 ± 4.26	79.63 ± 4.47	0.074
H/H	28.52 ± 0.93	26.42 ± 0.95	0.163

AHO: Australian White × Small-tailed Han × Oura-type Tibetan three-way crossbred sheep; O: Oura-type Tibetan sheep. SFA (saturated fatty acid), MUFA (monounsaturated fatty acid), PUFA (polyunsaturated fatty acid). The n-3 PUFA includes C18:3n-3, C20:3n-3, C20:5n-3, and C22:6n-3, while n-6 PUFA comprises C18:2n-6c, C18:3n-6, and C20:3n-6. The IA is calculated as [C12:0 + (4 × C14:0) + C16:0]/(PUFA + MUFA). The IT is determined by (C14:0 + C16:0 + C18:0)/[(0.5 × MUFA) + (0.5 × n-6) + (3 × n-3) + (n-3/n-6)]. The H/H is given by (C18:1 + ΣPUFA)/(C12:0 + C14:0 + C16:0). ^a,b^ different letters indicate significant differences between two groups (*p* < 0.05). Data were presented as means ± SEM.

Subsequently, we analyzed the amino acid composition of the lambs’ LTL muscle. Results were represented in Table 5. The AHO group exhibited a significant increase in glutamic (Glu) and methionine (Met) levels compared to the O group (*p* < 0.05). There was no significant difference in the levels of other amino acids between the O and AHO groups (*p* > 0.05).

**Table 5:** Amino acid composition in the LTL muscle of O and AHO lambs.

Type	Amino acid (%)/Taste description	O	AHO	*p*-Value
EEA	Threonine (Thr)/sweet	0.88 ± 0.03	0.87 ± 0.01	0.803
	Valine (Val)/bitter	0.99 ± 0.06	0.92 ± 0.05	0.415
	Isoleucine (Iie)/bitter	0.90 ± 0.03	0.91 ± 0.01	0.729
	Leucine (Leu)/bitter	1.57 ± 0.03	1.58 ± 0.02	0.834
	Phenylalanine (Phe)/bitter	0.73 ± 0.03	0.72 ± 0.02	0.843
	Lysine (Lys)/bitter (fresh)	1.84 ± 0.06	1.83 ± 0.02	0.960
NEAA	Aspartic acid (Asp)/fresh	1.74 ± 0.06	1.72 ± 0.03	0.811
	Serine (Ser)/sweet	0.76 ± 0.02	0.75 ± 0.01	0.833
	Glutamic acid (Glu)/fresh	3.04 ± 0.04^b^	3.18 ± 0.03^a^	0.036
	Proline (Pro)/sweet	0.52 ± 0.02	0.54 ± 0.01	0.439
	Glycine (Gly)/sweet	0.82 ± 0.01	0.80 ± 0.02	0.555
	Alanine (Ala)/sweet	1.11 ± 0.04	1.09 ± 0.02	0.739
	Methionine (Met)/bitter	0.35 ± 0.01^b^	0.45 ± 0.02^a^	0.003
	Tyrosine (Tyr)/bitter	0.66 ± 0.03	0.65 ± 0.03	0.693
	Histidine (His)/bitter (sweet)	0.85 ± 0.06	0.81 ± 0.03	0.511
	Arginine (Arg)/bitter	1.23 ± 0.03	1.22 ± 0.02	0.882

AHO: Australian White × Small-tailed Han × Oura-type Tibetan three-way crossbred sheep; O: Oura-type Tibetan sheep. EEA: essential amino acids, NEAA: nonessential amino acids. ^a,b^ different letters indicate significant differences between two groups (*p* < 0.05). Data were presented as means ± SEM.

We also analyzed the nucleotide content responsible for taste in the LTL muscle of lamb (Table 6). The LTL muscles in the AHO group exhibited a trend towards higher inosine monophosphate (IMP) levels compared to the O group (*p* = 0.072). There were no significant differences (*p* > 0.05) in the levels of guanosine monophosphate (GMP), 6-hypoxanthine, and inosine between the O and AHO groups.

**Table 6:** Nucleotide content related to taste in the LTL muscle of O and AHO lambs.

Ribonucleotide content (mg/100 g)	O	AHO	*p*-value
IMP	94.75 ± 1.93	138.79 ± 18.06	0.072
GMP	7.69 ± 0.05	7.47 ± 0.50	0.754
6-Hypoxanthine	36.93 ± 6.18	28.26 ± 4.48	0.271
Inosine	171.42 ± 16.67	161.80 ± 6.69	0.582

AHO: Australian White × Small-tailed Han × Oura-type Tibetan three-way crossbred sheep; O: Oura-type Tibetan sheep. IMP: inosine monophosphate; GMP: guanosine monophosphate. ^a,b^ different letters indicate significant differences between two groups (*p* < 0.05). Data were presented as means ± SEM.

### 3.4 Transcriptomic analysis of DEGs in LTL muscle and RT-qPCR validation

**Figure 1:**
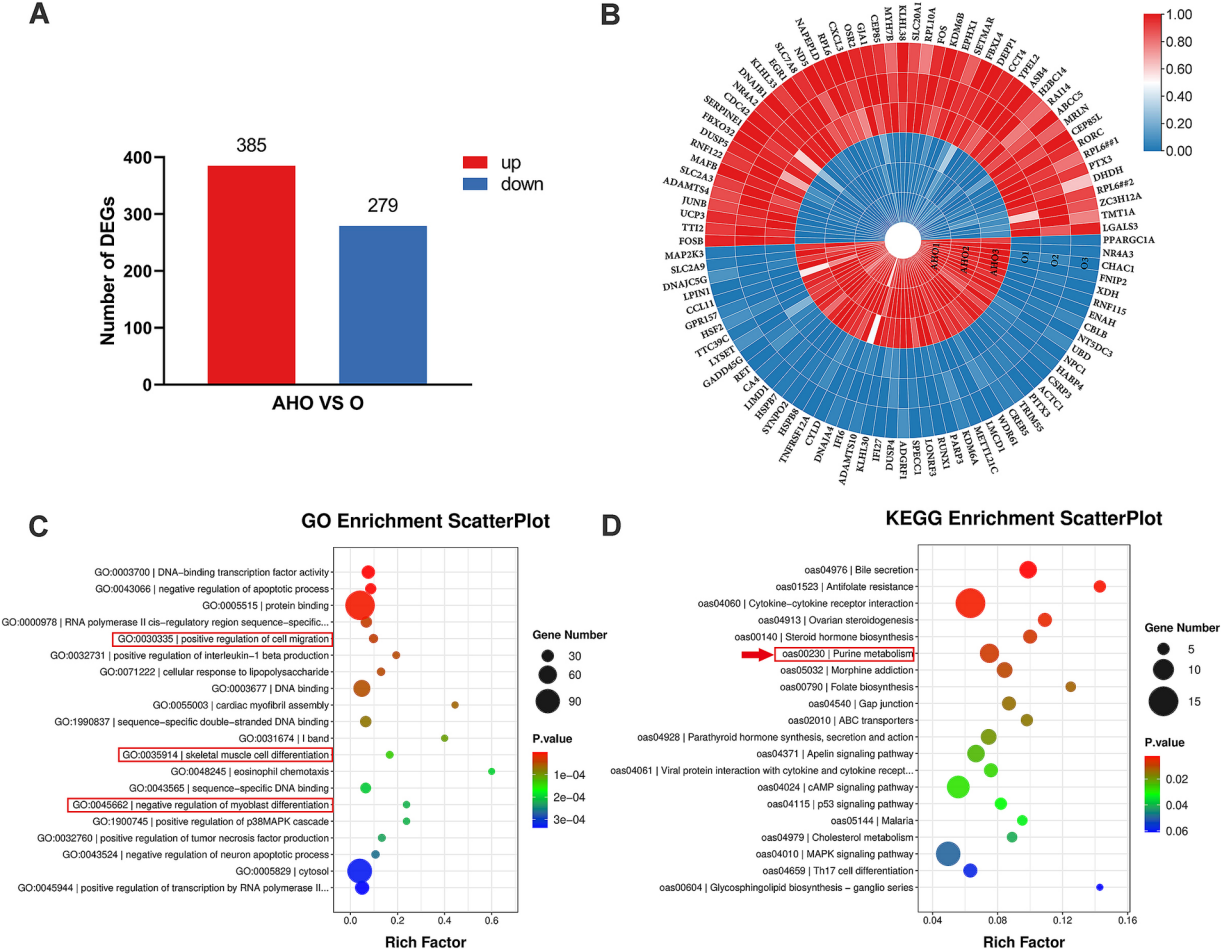
Transcriptomic sequencing and analysis of LTL muscle in Oura-type Tibetan lamb (O) and three-way crossbred lamb (AHO). **(A)** The histogram of the number of DEGs (*q* < 0.05 and fold change ≥ 2). **(B)** Heatmap illustrating the top 100 differentially expressed genes. The color shift from blue to red suggests relatively high expression levels of the genes. **(C)** The top 20 GO terms were identified through enrichment analysis, ranked by ascending *p*-value. **(D)** The top 20 KEGG pathway terms were identified through enrichment analysis, ranked by ascending *p*-values.

In depth, we were intrigued about investigating the underlying molecular process that accounts for the differences in performance and meat quality between the two groups. The transcriptomic analysis showed that a total of 664 DEGs were identified (*q* < 0.05 and fold change ≥ 2), of which 385 were upregulated and 279 were downregulated (Figure 1A). Figure 1B highlighted the top 50 DEGs with significant upregulation and downregulation between the two groups of lambs, including *PPARGC1A*, *CSRP3*, *LMCD1*, and *UCP3*.

GO and KEGG pathway enrichment analyses were conducted to clarify the functions of the DEGs. Figure 1C highlighted the top 20 significantly enriched GO terms. The top 5 enriched GO terms were “DNA-binding transcription factor activity,” “negative regulation of apoptotic process,” “protein binding,” “RNA polymerase II cis-regulatory region sequence-specific DNA binding,” and “positive regulation of cell migration.” Terms of “positive regulation of cell migration,” “skeletal muscle cell differentiation,” and “negative regulation of myoblast differentiation,” which associated with muscle development were also involved. Figure 1D showed the top 20 significantly enriched KEGG pathway terms. The top 5 enriched terms were “bile secretion,” “antifolate resistance,” “cytokine-cytokine receptor interaction,” and “ovarian steroidogenesis,” and “steroid hormone biosynthesis.” The “purine metabolism” pathway were also included.

**Figure 2:**
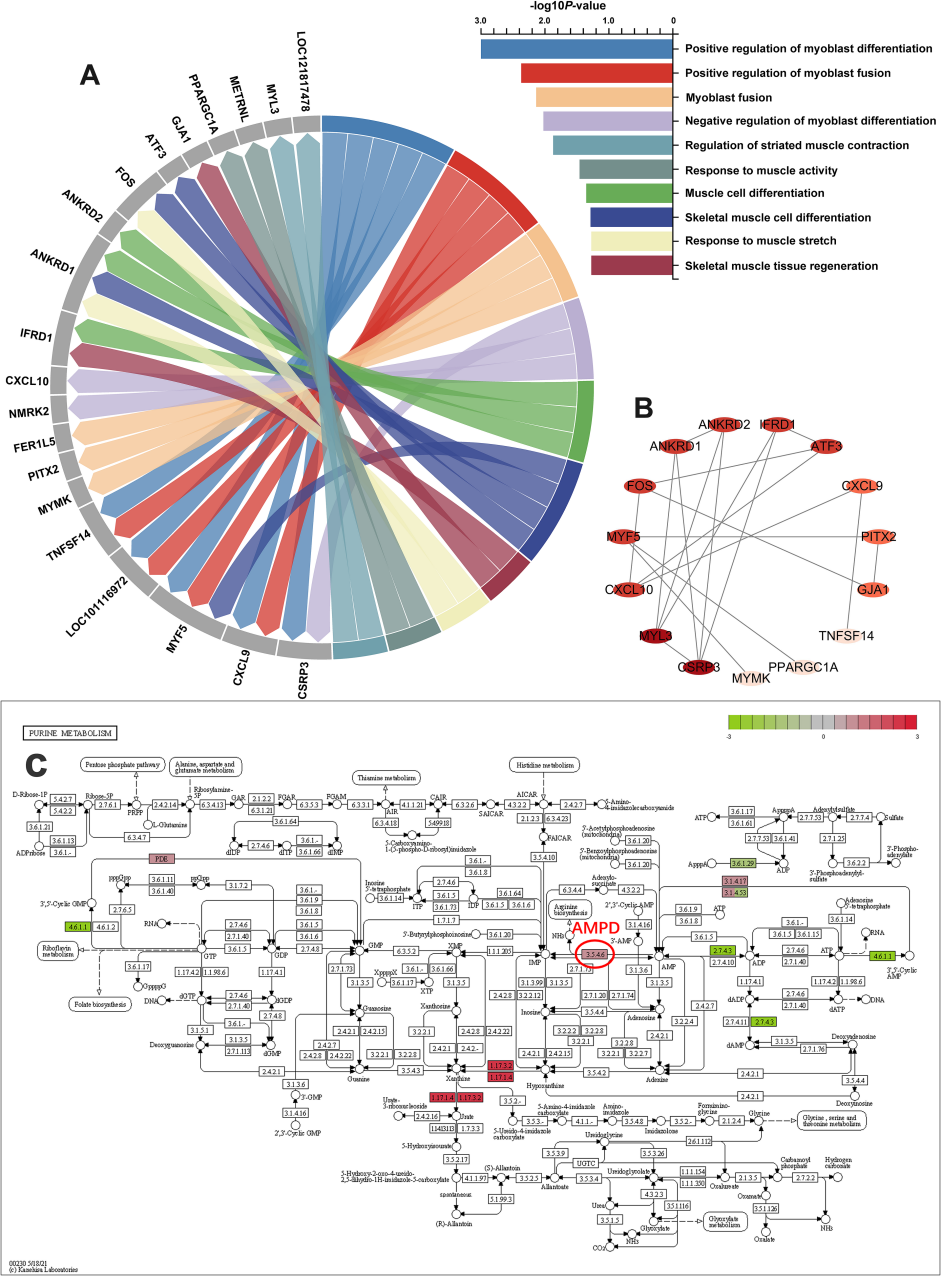
Hub DEGs associated with skeletal muscle development and meat flavor. **(A)** Identity and GO analysis of the candidate DEGs related to skeletal muscle development. **(B)** The gene-action network of the candidate DEGs. The network was constructed using STRING database and Cytoscape software, and the node with the greater degree of correlation among the candidate DEGs had a darker hue. **(C)** Overview of purine metabolism pathway (40). The color green indicates downregulated genes, and the color red indicates upregulated genes.

We further summarized all differential GO terms related to skeletal muscle development and their related DEGs, as shown in Figure 2A. A cluster of 20 hub DEGs was identified based on 10 related GO terms. These DEGs include *ANKRD1*, *ANKRD2*, *ATF3*, *CSPR3*, *CXCL9*, *CXCL10*, *PPARGC1A*, *FER1L5*, *FOS*, GJA1, *IFRD1*, *METRNL*, *MYF5*, *MYL3*, *MYMK*, *NMRK2*, *PITX2*, *TNFSF14*, LOC121817478, and LOC101116972. These genes were further analyzed using gene-action network analysis using Cytoscape software. After analysis with the network analyzer plugin, 20 DEGs were ranked according to their importance in the DEGs cluster, with genes having a higher degree of correlation among the candidate DEGs represented by a darker hue (Figure 2B). To illustrate the connection between DEGs and pathways, we also annotated the changes in DEGs in the KEGG pathway diagram. Figure 2C summarized the purine metabolism pathway, the hub of 3.5.4.6 (adenosine monophosphate deaminase, *AMPD*), which is upstream of IMP, showed significantly increased.

**Figure 3:**
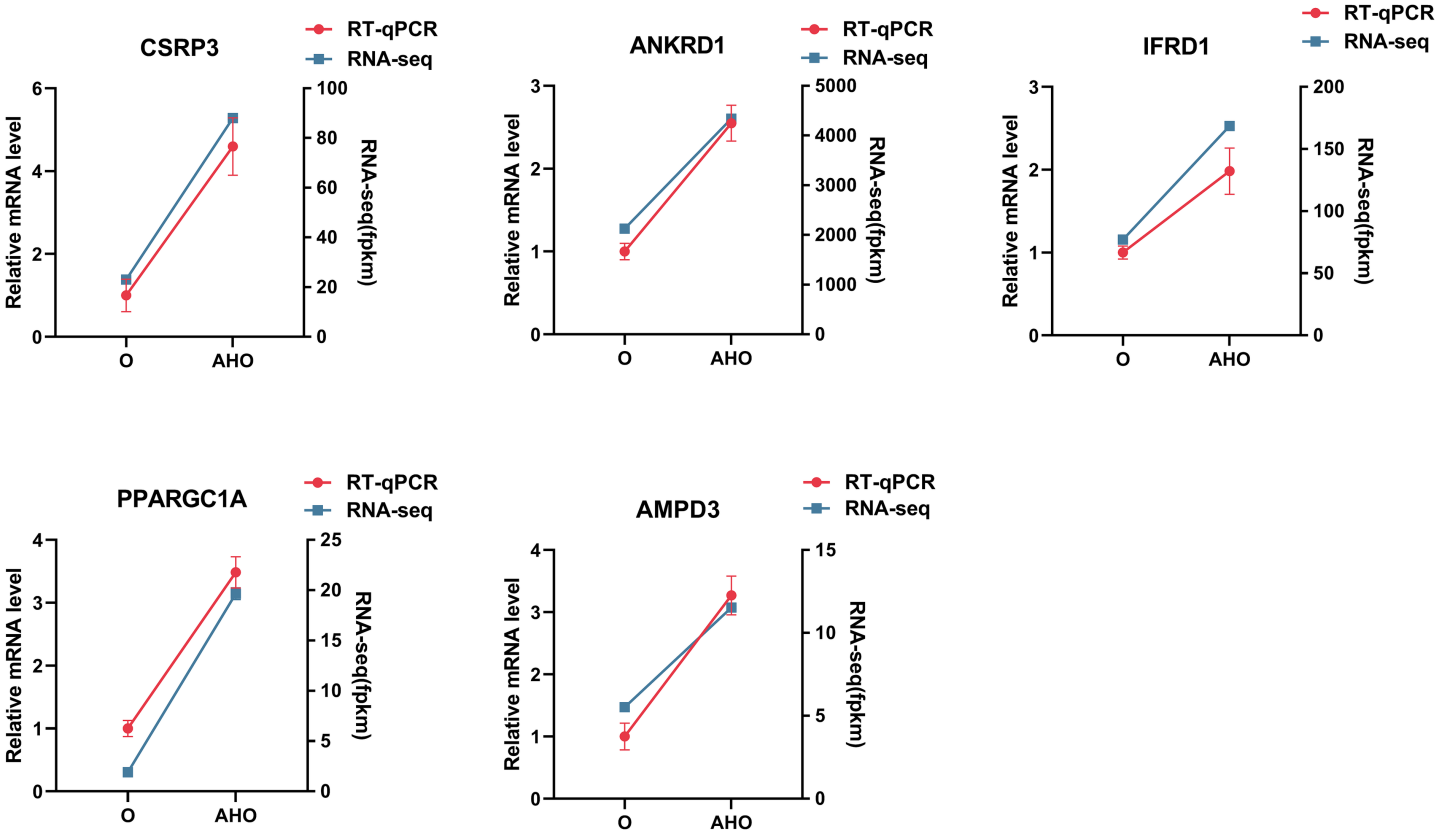
Comparison of DEGs between RT-qPCR and RNA-Seq results. The blue line represents RNA-Seq results, while the red bars show RT-qPCR data.

To confirm the reliability of transcriptomic results, five hub DEGs were selected for RT-qPCR validation, including *CSPR3*, *ANKRD1*, *IFRD1*, *PPARGC1A*, and *AMPD3*. Notably, transcriptome profiling demonstrated that these DEGs were significantly upregulated in the AHO group relative to the O groups. Consistently, the RT-qPCR results also showed that the expression levels of these genes were all significantly increased in the AHO group compared to the O group, confirming the accuracy of the transcriptome results (Figure 3).

## 4 Discussion

In this study, we found the Tibetan three-way crossbred lambs (AHO) showed better growth performance than the Tibetan lambs 5.5 months. It indicated that AHO lambs might benefit from hybrid vigor, thus enhancing their growth potential. This is consistent with previous studies showing that crossbred sheep generally demonstrated higher growth rates and weight gain compared to purebred sheep (20). The weights of the lambs at 0 months showed no significant difference, which might be related to the fact that the genetic advantages of hybrid sheep are not fully demonstrated in the early breeding stage. The enhanced live weight corresponds to improved overall body development, as seen in parameters such as body length, cannon circumference in 5.5 months. On the other hand, in terms of carcass traits, crossbreeding also yielded notable benefits. The AHO lambs exhibited a significantly higher carcass weight compared to Tibetan sheep, indicating a greater overall yield. The loin eye area, an important indicator of muscle development, was markedly larger in the crossbred sheep, suggesting that crossbred lambs are more favorable for meat production (4).

Then, we observed that evaluations of the LTL muscle meat quality showed no appreciable variations in pH, color, or cooking loss between the two groups, indicating that even though the AHO lambs are superior to growth rate than Tibetan lambs, these meat quality characteristics remained relatively stable, which is consistent with previous research indicating that crossbreeding does not always improve these meat quality traits (21).

Fatty acids in muscle are crucial for both enhancing meat flavor and serving as essential nutrients with significant physiological roles in the human body (22). This study identified 28 fatty acids, including 8 PUFAs, in the LTL muscle. As beneficial fatty acids for human, PUFAs play a variety of functions within biological systems, including resisting cardiovascular diseases and promoting growth and development (23). Higher PUFA contents also help improve meat taste (24). The study observed an increasing trend in total PUFA and n-6 PUFA levels within the AHO group. Linoleic acid (LA, C18:2n-6), and *α*-linolenic acid (ALA, C18:3n-3) are essential PUFAs that humans cannot synthesize (25). Reviews of existing evidence indicate that higher dietary intake or tissue levels of LA or ALA are linked to a lower incidence of metabolic syndrome, type 2 diabetes, and cardiovascular diseases (26, 27). The increasing trend in LA and ALA in the LTL muscle of crossbred lambs suggests an enhancement in meat nutritional quality. The PUFA/SFA ratio, atherogenic index (IA), thrombogenic index (IT), and H/H are commonly utilized to evaluate the impact of fatty acids on cardiovascular health, thromboprophylaxis, and atherosclerosis (28). Foods with a higher PUFA/SFA ratio are considered more beneficial for health (9). IT characterizes the thrombogenic potential of fatty acids, indicating their tendency to form blood clots in blood vessels, and foods in low IT is beneficial for avoiding cardiovascular diseases (29). The observed increase tendency in PUFA/SFA ratio and decrease in IT in the LTL muscle of crossbred lambs suggests an improvement in nutritional quality. However, although PUFA increased trend in the AHO group, the value of H/H ratio was slightly lower than that in the O group (*p* > 0.05), which may be due to the increase trend of SFA.

We further determined the contents and types of amino acids in the lamb of the two groups. It has been demonstrated that the nutritional value of meat increases with its essential amino acid content (25). In this study, we observed that the content of the two essential amino acids, Met and Glu in the muscles of the AHO group increased significantly than that of the O group.

In addition to the nutritional quality, we further investigated whether the crossbreeding has an impact on the umami taste of sheep. The amino acid composition of muscle significantly influences the nutritional quality of meat and affects its flavor (30). Diverse amino acids possess different tastes during cooking. Umami taste, characterized as meaty, savory, brothy, or delicious, is activated by flavor-enhancing non-volatile compounds (31). Glu, Arg, Asp., and Gly amino acids provide umami taste, Gly, Ser, Thr, Lys, Pro, and Ala amino acids provide a sweet taste, and Leu, Ala, and Arg amino acids provide aromatic flavor to the meat (32). In this study, we found the content of Glu in the muscles of the AHO group increased significantly.

On the other hand, we focused on IMP, another major umami substance, which is widely regarded as an important indicator for evaluating the umami taste of meat (33). In addition, meat that contains too much inosine and hypoxanthine, which are IMP metabolites, will taste bitter (34). Our findings indicated a tendency for higher IMP content in the LTL muscle of AHO lambs compared to O lambs. However, IMP content in the LTL muscle is not statistically significant, which may be related to the limited sample size in this study. Meanwhile, the contents of inosine and 6-hypoxanthine in LTL muscle were consistent between the two groups. Combining with the findings in amino acids and nucleotides of LTL muscle, the result indicated that the meat of crossbred lambs may have better consumer satisfaction on meat, which is consistent with a previous study that also compared the taste of crossbred lamb meat with purebred lamb (20).

More deeply, we were curious to investigate the underlying molecular mechanism that account for the variations, in terms of body conformation, carcass traits and meat quality in the two groups. Transcriptomic analysis identified DEGs in the LTL muscle of two lamb groups. Our study concentrated on DEGs in the LTL muscle associated with muscle development and flavor. Among the 20 candidate DEGs related to muscle development, *CSRP3*, *ANKRD1*, *IFRD1*, and *PPARGC1A* were genes with high expression levels and high fold change in LTL muscle. These genes are crucial for muscle development and the structural integrity of muscle cells.

Previous research demonstrated that reduced *CSRP3* expression inhibits chicken satellite cell differentiation (35). *ANKRD1* was identified as a potential regulator of muscle cell development. Its knockdown decreased the proliferation but increased the differentiation of C2C12 myoblasts (36). *IFRD2* overexpression promoted bovine muscle-derived satellite cell differentiation (37). Ma et al. reported that *PPARGC1A* could activate slow-twitch muscle phenotype and induce muscle hypertrophy (38). For meat flavor DEGs, AMPD catalyzes the hydrolytic deamination of adenosine monophosphate to IMP in skeletal muscle. The *AMPD3* gene encodes a member of the AMP deaminase gene family, affects the production of an AMPD holoenzyme of varying subunit composition in myocytes (39). The study found a notable increase in *AMPD3* gene expression in the LTL muscle of AHO group lambs. The variations in *AMPD3* between the two groups may be linked to differing IMP levels in AHO and O lambs, with a trend of increased IMP observed in the LTL muscle of crossbred lambs. However, further in-depth study is needed to verify this phenomenon. Based on these results, we are now planning large-scale F2 generation crossbred experiments on the Qinghai-Tibet plateau, where this crossbred approach could significantly improve production efficiency under local conditions.

## 5 Conclusion

In summary, our study revealed that Tibetan three-way crossbred lambs (AHO) exhibited superior growth performance and carcass traits and higher meat quality. The genes of *CSRP3*, *ANKRD1*, *IFRD1*, *PPARGC1A*, and *AMPD3* had close correlations with muscle development and muscle flavor, and they could be considered as potential candidates for promoting muscle development or improving the IMP content in AHO lamb. The crossbreeding of Tibetan sheep potentially offers an efficient way to produce high-quality lamb products, and may serve as a promising genetic resource for sustainable lamb production systems in high-altitude environments of Qinghai-Tibetan plateau. In view of the limitations of this study, we will conduct longer-term feeding experiments with larger animal groups in subsequent research to better and more scientifically explore the specific growth patterns and meat quality of AHO sheep.

## Data Availability

The datasets presented in this study can be found in online repositories. The names of the repository/repositories and accession number(s) can be found below: https://www.ncbi.nlm.nih.gov/, PRJNA1187451.

## Footnotes

^1^ http://www.geneontology.org/

^2^ http://www.genome.jp/kegg/

^3^ https://www.omicstudio.cn/tool

## Glossary

ALA: *α*-linolenic acid
AMPD3: adenosine monophosphate deaminase 3
ANKRD1: ankyrin repeat domain-containing protein 1
ATF3: activating transcription factor 3
CSRP3: cysteine and glycine-rich protein 3
CXCL9: C-X-C motif chemokine ligand 9
DEGs: differentially expressed genes
FER1L5: fer-1 like family member 5
FOS: fos proto-oncogene
GJA1: gap junction protein alpha 1
Glu: glutamic acid
GMP: guanosine-5′-monophosphate
H/H: hypocholesterolemic/hypercholesterolemic ratio
IA: index of atherogenicity
IFRD1: interferon-related developmental regulator 1
IMP: inosine-5′-monophosphate
IT: index of thrombogenicity
LA: linoleic acid
LMCD1: LIM and cysteine-rich domains 1
Met: methionine
METRNL: meteorin like
MUFA: monounsaturated fatty acid
MYF5: myogenic factor 5
MYL3: myosin light chain 3
MYMK: myomaker
NMRK2: nicotinamide riboside kinase 2
PITX2: paired like homeodomain 2
PPARGC1A: peroxisome proliferator-activated receptor gamma coactivator 1 alpha
PUFA: polyunsaturated fatty acid
SFA: saturated fatty acid
TNFSF14: tumor necrosis factor superfamily member 1
UCP3: uncoupling protein 3
