# Supplementary material for: A comparative analysis of carcass traits and meat quality between Tibetan sheep and their three-way crossbred sheep
Source: Front Nutr. 2025 Jul 16;12:1620180. doi: 10.3389/fnut.2025.1620180 (PMC12307410; doi:10.3389/fnut.2025.1620180)
Supplement: Supplementary file 1 [file Table_1.DOCX]

Supplementary Material

# Supplementary Tables

Table S1. Sequences of genes-special primers used for RT-qPCR.

| Genes | Description | Primer sequences (5'-3') |
| --- | --- | --- |
| AMPD3 | Adenosine monophosphate deaminase 3 | F: ATGCAAAGCAGAGACCTCCCAC |
|  |  | R: AGATGTTCAGCTTGGGGAATTGG |
| ANKRD1 | Ankyrin repeat domain 1 | F: AGCCCAGATCGAATTCCGTG |
|  |  | R: GCGGTGCTGAGCAACTTATC |
| CSPR3 | Cysteine and glycine rich protein 3 | F: TGCGGAAGAAATCCAGTG |
|  |  | R: AGCCAGCACCTTGTCCATAC |
| PPARGC1A | Peroxisome proliferator-activated receptor gamma coactivator 1 alpha | F: CACCAGTGGACACGAGGAAA |
|  |  | R: TGGAGGAGGGGGCATCTTTA |
| IFRD1 | Interferon-related developmental regulator 1 | F: GGCATGTACCAGGAAGCAGT |
|  |  | R: CTCAGTTGGTGCCTGGGTAT |
| GAPDH | Glyceraldehyde-3-phosphate dehydrogenase | F: TGTTTGTGATGGGCGT |
|  |  | R: TCTGGGTGGCAGTGAT |

Table S2. The meat quality characteristics in *longissimus dorsi* muscle of O and AHO lambs.

| Item | O | AHO | *P*-Value |
| --- | --- | --- | --- |
| pH (45 min) | 6.25 ± 0.10 | 6.38 ± 0.13 | 0.457 |
| pH (24 h) | 5.88 ± 0.16 | 5.87 ± 0.19 | 0.970 |
| Meat color *a* | 8.91 ± 1.18 | 8.97 ± 0.75 | 0.962 |
| Meat color *b* | 6.59 ± 0.86 | 7.48 ± 0.61 | 0.413 |
| Meat color *L* | 28.23 ± 1.76 | 30.19 ± 1.33 | 0.394 |
| Cooking loss (%) | 43.85 ± 1.73 | 44.62 ± 0.71 | 0.666 |
| Crude protein (%) | 19.43 ± 0.62 | 19.36 ± 0.43 | 0.932 |
| Crude fat (%) | 5.53 ± 0.07 | 3.98 ± 0.68 | 0.104 |

Note: Data were represented as means ± SEM.
